# Point: Antenatal corticosteroid treatment in the late preterm period—Are the benefits worth the potential risks?

**DOI:** 10.1111/ppe.12927

**Published:** 2022-09-29

**Authors:** Eero Kajantie

**Affiliations:** ^1^ Population Health Unit Finnish Institute for Health and Welfare Helsinki and Oulu Finland; ^2^ Clinical Medicine Research Unit, MRC Oulu University of Oulu and Oulu University Hospital Oulu Finland; ^3^ Department of Clinical and Molecular Medicine Norwegian University of Science and Technology Trondheim Norway; ^4^ Children's Hospital Helsinki University Hospital and University of Helsinki Helsinki Finland

Antenatal corticosteroid treatment (ACT) is one of the success stories in improving the outcome of babies born preterm. There is robust evidence across high, middle and low resource settings that the treatment is effective in preventing respiratory distress syndrome and perinatal and neonatal death.^1^ While there are uncertainties in the optimal dosing regimen, repeated treatments and possible contraindications, guidelines are consistent in recommending the treatment up to 34 weeks of gestation^2^, ^3^, ^4^, ^5^, ^6^ at least in settings where gestational age can be accurately assessed and adequate childbirth and neonatal care are available.^6^

**TABLE 1:** Select guidelines on antenatal corticosteroid treatment (ACT) in the late preterm period, 34^0/7^–36^6/7^ weeks

Guideline and year of most recent update	Recommendation on ACT in the late preterm period, 34 + 0 to 36^6/7^ weeks	Other points
WAPM‐PMF 2022^2^ FIGO 2021^3^	Not routinely recommended Should not be offered routinely. Should be considered in light of the balance of risks and benefits for individual women	Specific advice against ‘just in case’ treatment for high‐risk women (treatment should be given only when preterm birth is expected within ≤7 days)
European Consensus Guidelines 2019^4^	Not currently recommended for women in spontaneous preterm labour	This late preterm recommendation included in the introductory text of the point‐by‐point ACT recommendations, which themselves do not take stance to the late preterm period Previous 2016 guideline recommended that ACT may be considered in the late preterm period provided there is no evidence of chorioamnionitis
ACOG 2017^5^	Recommended for women who have not received a previous course of ACT	
WHO 2015^6^	Should not be routinely administered when gestational age is suspected to be more than 34 weeks Not recommended in women undergoing planned caesarean section at late preterm gestations	ACT in general recommended in settings where gestational age can be accurately determined and adequate childbirth and preterm newborn care is available. In variance with most other guidelines, ACT not recommended when there are clinical signs of maternal chorioamnionitis

*Note*: With some difference in detail, all these guidelines recommend antenatal corticosteroid treatment at least up to 34 weeks. Guidelines are presented in the order of the publication of the most recent update.

Abbreviations: ACOG, American College of Obstetricians and Gynaecologists; ACT, Antenatal corticosteroid treatment; FIGO, International Federation of Gynaecology and Obstetrics; WAPM‐PMF, World Association of Perinatal Medicine and Perinatal Medicine Foundation; WHO, World Health Organisation.

There has been much more controversy in whether the treatment indications should be expanded to include imminent late preterm birth, from 34 to 36 completed weeks of gestation. Currently most major clinical guidelines do not recommend routine treatment during this period (Table 1). The scenario is different: while late preterm infants do have a higher risk of respiratory distress syndrome and higher perinatal and neonatal mortality than infants born at term, these risks are much smaller than in infants born earlier in gestation. Generally, it is only feasible to target respiratory distress as an outcome as baseline levels of mortality and other severe outcomes are low. Moreover, including these gestational weeks to treatment indications would lead to substantial increases in numbers of treatment‐exposed children; compared with infants born before 34 weeks, the rate of late preterm birth is more than 2.5‐fold greater. This highlights the need to carefully quantify and balance the risks and benefits.

Hutcheon and Liauw^7^ make an important contribution to this debate. They target a key limitation of randomised controlled trials (RCTs): trial participants frequently differ from real‐world populations. Much of the evidence that has argued for ACT during the late preterm period comes from the Antenatal Late Preterm Steroid (ALPS) trial conducted in the USA 2010 to 2015.^8^ ALPS recruited women with imminent preterm birth between 34^0/7^ and 36^6/7^ weeks. The need for neonatal respiratory support was reduced from 14.4% in the control group to 11.6% in the ACT group. However, women were recruited in ALPS on average earlier in gestation than all women presenting to a hospital for delivery in the late preterm period, making their infants more likely to have respiratory distress and thus benefit from ACT, compromising the external validity of the finding. Hutcheon and Liauw use a simple weighting process to improve the generalisability of the finding to the trial source population and transportability to different populations. This reduced the 2.8 (95% confidence interval [CI] 0.3, 5.3) percentage point absolute risk reduction to 2.2 (95% CI 0.0, 4.6) percentage points and increased the number needed to treat to prevent one case of respiratory support from 35 to 46. Of note, mortality at these gestational weeks is low (2/2827 ALPS children died in the neonatal period) making any possible mortality benefit small and practically unfeasible to find.

These numbers should be weighed against the possible harms of ACT. While prenatal glucocorticoid exposure in animal experiments can have lifelong consequences on neurodevelopment and metabolism, few if any long‐term harm has been demonstrated in RCT follow‐up studies. However, these studies have limited power to assess long‐term secondary outcomes and thus might give a false sense of security. Concern was raised by a recent whole‐population register study from Finland finding that 12.0% of ACT‐exposed children had been diagnosed with any mental or behavioural disorder, compared with 6.5% of those not exposed. This corresponded to an absolute risk increase 5.56% (95% CI, 5.04, 6.19), and a number needed to harm of 18 (95% CI 16–20).^9^ The associations persisted after confounder adjustment and in within‐sibpair comparisons.^10^

As to limitations, Hutcheon and Liauw mention that real‐world ACT administration may include more suboptimally timed administration, which they could not assess with the available data. They argue that the practical impact of this may be minor.^7^ However, an important feature that may affect the transportability to real‐world populations that they were not able to target was the proportion of infants actually born preterm. Trials may prefer to recruit women with highest risk of preterm delivery. In the ALPS trial recruiting between 34^0/7^ and 36^6/7^ weeks, 16.4% ended up being born at term, from 37^0/7^ weeks onwards. This can be very different in a real‐world population: in the Finnish whole‐population register study, during an era when treatment was recommended up to 34^6/7^ weeks, almost half (45.3%) of treatment‐exposed infants ended up being born at term.^9^ These infants are unlikely to benefit from any advance in foetal maturation, a desired mechanism of ACT treatment. Accordingly, in that study the relative risks of adverse outcomes associated with ACT exposure were largely confined to ACT‐exposed infants who ended up being born at term.^9^ The difficulty of predicting preterm birth has been recognised for example in the 2021 recommendation of International Federation of Obstetrics and Gynaecology FIGO that specifically advices against ‘just in case’ ACT treatments.^3^

One of the key challenges in many perinatal medicine questions including ACT is transportability across settings—a frequent question in practice is to what extent results obtained from a high‐resource setting can be generalised to settings with lower resource levels. In such scenarios the assumptions for transportability, also discussed by Hutcheon and Liauw,^7^ may be more challenging to meet, and high‐quality evidence would require RCTs conducted in a similar setting. That said, the ALPS trial provides a nicely illustrative example of a study where weighting for a simple variable, in this case gestational age, can be used to improve the external validity of the treatment effects of an RCT in a relatively similar population.

## About the author

**Eero Kajantie** is specialist physician in Pediatrics, Clinical Genetics and Public Health. He serves as Professor of Lifecourse Medicine at University of Oulu and Research Manager at Finnish Institute for Health and Welfare and is also affiliated at Norwegian University of Science and Technology and University of Helsinki. His main research interests are child and adult outcomes of pregnancy disorders, in particular preterm birth. His research spans from whole‐population register studies to carefully phenotyped longitudinal cohorts in high and low resource settings and several international consortia.

## FUNDING INFORMATION

The author has received grant support from Academy of Finland, European Commission (RECAP Preterm 733280), Finska Läkaresällskapet, Foundation for Cardiovascular Research, Foundation for Pediatric Research, Novo Nordisk Foundation, Signe and Ane Gyllenberg Foundation, Sigrid Juselius Foundation and Yrjö Jahnsson Foundation to study the long‐term outcomes of preterm birth and related traits including antenatal corticosteroid use.

## CONFLICT OF INTEREST

The author has received grant support from Academy of Finland, European Commission (RECAP Preterm 733,280), Finska Läkaresällskapet, Foundation for Cardiovascular Research, Foundation for Paediatric Research, Novo Nordisk Foundation, Signe and Ane Gyllenberg Foundation, Sigrid Juselius Foundation and Yrjö Jahnsson Foundation to study the long‐term outcomes of preterm birth and related traits including antenatal corticosteroid use.

## Data Availability

This commentary used no data.
